# Tolerogenic dendritic cells in type 1 diabetes: no longer a concept

**DOI:** 10.3389/fimmu.2023.1212641

**Published:** 2023-06-14

**Authors:** Nick Giannoukakis

**Affiliations:** ^1^ Department of Pathology, University of Pittsburgh School of Medicine, Pittsburgh, PA, United States; ^2^ Department of Biological Sciences, Carnegie Mellon University, Pittsburgh, PA, United States

**Keywords:** type 1 diabetes, dendritic cells, tolerance, immunomodulation, autoimmunity

## Abstract

Tolerogenic dendritic cells (tDC) arrest the progression of autoimmune-driven dysglycemia into clinical, insulin-requiring type 1 diabetes (T1D) and preserve a critical mass of β cells able to restore some degree of normoglycemia in new-onset clinical disease. The safety of tDC, generated *ex vivo* from peripheral blood leukocytes, has been demonstrated in phase I clinical studies. Accumulating evidence shows that tDC act via multiple layers of immune regulation arresting the action of pancreatic β cell-targeting effector lymphocytes. tDC share a number of phenotypes and mechanisms of action, independent of the method by which they are generated *ex vivo*. In the context of safety, this yields confidence that the time has come to test the best characterized tDC in phase II clinical trials in T1D, especially given that tDC are already being tested for other autoimmune conditions. The time is also now to refine purity markers and to “universalize” the methods by which tDC are generated. This review summarizes the current state of tDC therapy for T1D, presents points of intersection of the mechanisms of action that the different embodiments use to induce tolerance, and offers insights into outstanding matters to address as phase II studies are imminent. Finally, we present a proposal for co-administration and serially-alternating administration of tDC and T-regulatory cells (Tregs) as a synergistic and complementary approach to prevent and treat T1D.

## Introduction

**Figure 1:**
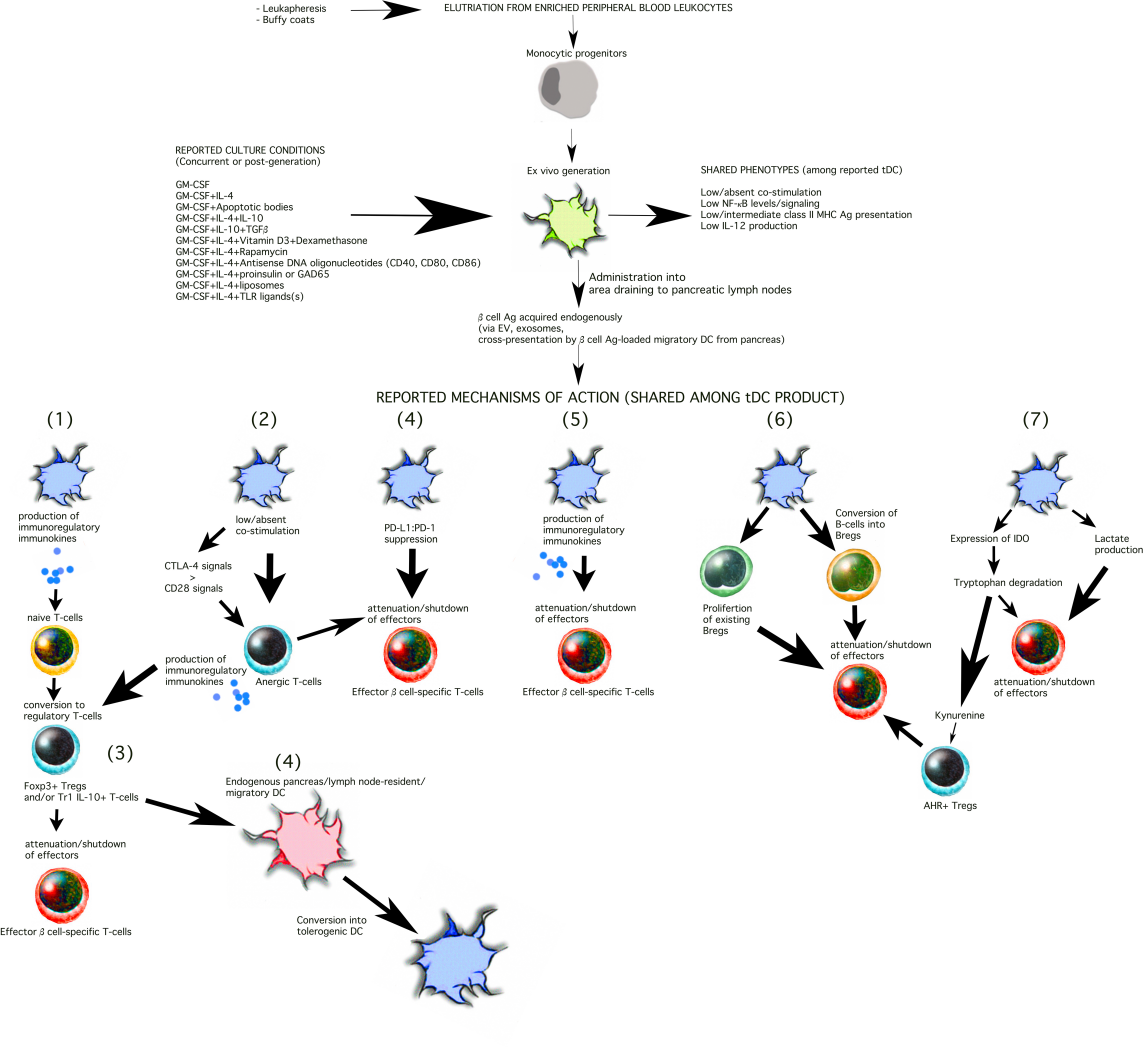
Shared phenotypes and mechanisms of action among T1D-targeting tDC. The current methods to generate tDC begin with peripheral blood leukocytes that are separated into monocytes either by traditional centrifugation methods or mechanical elutriation. The monocytes are then cultured ex vivo for 5-9 days towards differentiation into DC in the presence of either GM-CSF alone or with the additional of a number of other constituents illustrated in the Figure (top). These constituents are added into the differentiation cocktail at the start of the cell culture (e.g. IL-4, dexamethasone, vitamin D3, antisense DNA oligonucleotides targeting co-stimulation protein expression) and replenished during the generation period, or are added at the end of the differentiation period for an additional period of 1-5 days. While a universal purity marker for tDC has not yet been discovered, tDC clinical release criteria currently rely on low surface expression of CD86 and class II MHC (HLA) and low IL-12 production *in vitro* at the end of the cell generation process. The tDC have been administered i.v., s.c., or i.d. In some trials, the tDC have been administered into the abdomen at a site that drains, partially, to the pancreatic lymph nodes (13–15). Among the reported outcomes, verified in mouse models, tDC commonly re-establish and/or stimulate seven mechanisms of action as illustrated in the Figure 1) Conversion of naïve T-cells into regulatory T-cells that may belong to the Tr1 and/or Foxp3 T-cell populations. Tr1 and Foxp3+ induced Tregs are not mutually exclusive and may be β cell antigen-specific or polyclonal. tDC may achieve this by producing immunokines that convert T-cells into regulatory cells and/or via induction of anergic T-cells (mechanism 2, illustrated). Regulatory T-cells then suppress the activity or activation state of effector β cell autoreactive T-cells inside the lymph node space which they share with the tDC or inside the pancreas, close to the inflamed islets (mechanism 3, illustrated). While inside the pancreatic lymph nodes, tDC can also, on their own, or via regulatory T-cells, convert resident “immature/semi-mature” DC into tDC. β cell Ag can be acquired by the exogenously-supplied tDC in at least three possible ways: a) via extracellular vesicles draining from the inflamed pancreas; b) via cross-presentation/cross-dressing of Ag draining from the inflamed pancreas acquired by lymph node-resident “immature/semi-mature” DC; and/or c) migratory DC that arrive from inflamed pancreas carrying β cell-derived Ag and cross-presented/cross-dressed to tDC or endogenous tDC-”reprogrammed” lymph node-resident DC. Direct suppression of effector β cell autoreactive T-cells by the tDC (or tDC-directed, “reprogrammed” endogenous lymph node-resident DC) can additionally be achieved via PD-L1:PD-1 signaling (mechanism 4) and/or immunosuppressive immunokines produced by the tDC (mechanism 5, e.g. IL-10, TGFβ). A novel mechanism of action (mechanism 6) involves the differentiation of B-cells into Bregs and/or the proliferation of existing Bregs. Retinoic acid has been shown to be one of the tDC-produced molecules involved in the process. It is certain that other mechanisms inducing Bregs and/or that attenuate autoreactive B-cells remain to be discovered. Finally, in some instances, tDC express the enzyme IDO (mechanism 7, illustrated), which breaks down tryptophan, an amino acid necessary for T-cell survival and proliferation. Kynurenine, one of the breakdown products, can act as a ligand for the aryl hydrocarbon receptor (AHR), expressed in some regulatory T-cells to potentiate their suppressive capacity (mechanism 7).

With the recent news that Teplizumab (TZIELD™), a humanized CD3-targeting monoclonal antibody received FDA approval to delay the onset of Stage 3 type 1 diabetes mellitus (T1D) in adults and children =>8 years of age (1, 2), a media frenzy resulted in misrepresentation of the product’s actual efficacy and also misrepresented the careful comments and conclusions of scientists working with the antibody for more than 20 years. While the outcomes of important clinical studies over the past 10 years strongly support the efficacy of Teplizumab in a very selected population of pre-diabetic individuals (the formal characteristics of “responders” remain to be comprehensively defined (2–7);), T1D remains far from being cured and Teplizumab use has not resulted in an across-the-board delay or prevention of transition into Stage 3 or insulin-requiring diabetes (6, 8, 9). Thus, the search for a more comprehensive and more robust therapy remains to be discovered or developed. In this review, the case for tolerogenic dendritic cells (tDC) is once again [prior excellent reviews and expert opinion have already been published (10–12)] presented with more recent information that demonstrate that tDC act to re-enforce, re-make, and strengthen not one, but at least three interweaving immunosuppressive and immunoregulatory leukocyte networks. One can view tDC as the command center of a multi-level, multi-dimensional defense network (reviewed in (10–12) and illustrated in **Figure 1** that no other T1D treatment has yet achieved.

tDC human use has now matured and entered the same space as tumor-fighting DC therapy. **Table 1** presents the human clinical trials in the space of autoimmunity where safety outcomes have been reported. In all these studies, even those that are currently ongoing or recently completed, there were no reports of significant adverse events suggesting that the administration of the tDC could be well-tolerated, pending the anticipated reporting of the of the safety study outcomes.

**Table 1:** Human tDC clinical trials in the space of autoimmunity.

Condition	Study Phase	Study Type	tDC character	Study Status	Clinicaltrials.gov Identifier	Major Reported Outcomes	Reference(s)
T1D	II	Randomized, double blind, placebo-controlled, parallel	Monocyte-derived DC generated in the presence of a mixture of antisense oligonucleotides targeting CD80, CD86, CD40	In preparation (2023)	NCT02354911		
	I/II	Randomized, double-blind, placebo controlled, parallel	Monocyte-derived autologous dendritic cell therapy (AVT001)	Enrolling	NCT03895996		
	I	Non-randomized, single arm	Pro-insulin-loaded Vitamin D3-generated DC from monocytes	Completed	NTR5542		(15)
	I	Open label, pilot	Pro-insulin peptide (C19-A3)-loaded Vitamin D3-generated DC from CD14+ monocytes	Ongoing	NCT04590872		Nakamura, R: A pilot study to evaluate the safety and feasibility of autologous tolerogenic dendritic cells loaded with proinsulin peptide (C19-A3) in patients with type 1 diabetes; https://clinicaltrials.gov/ct2/show/NCT04590872.
	I	Randomized, double-blind, single arm	Monocyte-derived DC generated in the presence of a mixture of antisense oligonucleotides targeting CD80, CD86, CD40	Completed	NCT00445913	No adverse events identified, well-tolerated, detection of C-peptide in long-standing diabetics, increased frequency of B-cells exhibiting Breg characteristics. First ever clinical trial where tDC used in autoimmunity.	(13)
MS	I/IIA	Non-randomized, parallel assignment, open label	Vitamin D3-treated DC derived from monocytes and loaded with a pool of myelin peptides	Enrolling	NCT02618902		
	I/IIa	Non-randomized, parallel assignment, open label	Vitamin D3-treated DC derived from monocytes and loaded with a pool of myelin peptides	Enrolling	NCT02903537		
	I	Single arm, open Label	Dexamethasone-treated DC derived from monocytes and loaded with myelin peptides or aquaporin-4- derived peptide	Completed	NCT02283671	Treatment was safe and well-tolerated, decrease in frequency of CD8, NK, and CD14+ CD56+ cells in participants	(16)
RA	I	Randomized, parallel, open label	Dexamethasone+ Vitamin D3-treated DC derived from monocytes and loaded with autologous synovial fluid	Completed	NCT01352858	Treatment was safe and well-tolerated, Symptoms were improved in 2 patients at the highest cell dose	(17)
	I	Interventional, single arm, open label	DC derived from monocytes and loaded with pulsed with citrullinated filaggrin, and vimentin peptides	Completed	CRiSKCT0000035	Treatment was safe and well-tolerated, decreased number of of IFNγ - producing T cells and autoantibody titers	
	I	Non-randomized, control group, open label	NF-κB inhibitor-treated DC derived from monocytes and loaded with citrullinated RA-relevant peptides	Completed	Rheumavax	Treatment was safe and well-tolerated; decrease in effector T-cells frequency and T-effector:Treg ratio; reduced pro-inflammatory cytokine and chemokine concentration in serum; decrease in DAS28	(18)
	I	Single arm, open label	Dexamethasone-treated DC derived from monocytes	Completed	NCT03337165	Treatment was safe and well-tolerated, decrease in DAS28, improvement	(19)
Colitis	I	Sequential-cohorts, dose-range	Dexamethasone+ Vitamin A-treated DC derived from monocytes	Completed	2007-003469-42	3 participants showed positive clinical response, 3 withdrew due to worsening of symptoms	(20)
		Randomized, parallel, single blind	Dexamethasone-treated DC derived from monocytes	Terminated (low enrollment)	NCT02622763		
Transplantation	I/IIa Kidney transplantation	Single-arm, open label	GM-CSF-generated DC derived from monocytes	Completed	NCT02252055	Treatment was safe and well-tolerated	(21, 22)
	I Kidney transplantation	Non-randomized, Sequential, dose-escalation, open-label	Vitamin D3+IL-10 treated DC derived from monocytes	Enrolling	NCT03726307		
	I/II Liver transplantation	Non-randomized, prospective, open-label, non-controlled	Vitamin D3+IL-10 treated DC derived from monocytes	Active, not enrolling	NCT03164265		

The first symbol (↓) indicates a decrease in value of experimentral intervention from control values. The second symbol (↑) indicates an increase in value of experimentral intervention from control values.

T1D is the clinical outcome of a relapsing-remitting T-cell driven autoimmunity that, across three stages, gradually impairs and damages a mass of pancreatic β cells that requires exogenous insulin replacement to maintain normoglycemia (23, 24). While a combination of certain alleles at the *HLA*, *INS*, *PTPN22*, and *IL2RA* loci, together confer a substantial risk to develop the disease (25–27), there are many individuals who, in spite of inheriting high-risk alleles at one or more of these major loci, remain diabetes-free, even though evidence indicates that β cell-targeting autoantibodies and T-cells can be detected in the circulation many years prior to clinical onset (23). Mechanistically, the immunological basis of the relapsing-remitting T-cell-driven inflammation inside the pancreas is failure of central tolerance, where β cell-specific thymocytes are not deleted, as well as peripheral loss of tolerance to β-cell proteins such as insulin, glutamic acid decarboxylase 65 (GAD65), insulinoma-associated-2 (IA-2), and Zinc Transporter T8 (28, 29). T-cells reactive to these major β cell proteins are detected in the pancreata of newly-diagnosed humans as well as inside the pancreas of the NOD/LtJ mouse strain even weeks before the onset of clinical hyperglycemia (30–35). Pancreas-resident DC with markers associated with tolerogenic phenotype have been discovered, and their frequency disappears with the progression of T-cell infiltration around and into the pancreatic islets of Langerhans (33–40). This supports the hypothesis that, pancreas-resident tDC normally enforce immunoregulation (41–50) via maintenance of peripheral tolerogenic lymphocyte networks and their impairment/disappearance is permissive for the autoreactive T-cells to be unleashed around and inside the islets of Langerhans. This hypothesis, and its accumulating evidence in mouse, rat, and human tissues, motivated the consideration to use tDC to re-establish these immunoregulatory networks, or to create new networks of regulatory cells whose functional outcome would be some form of restored tolerance, inside newly-diagnosed T1D in order to “reverse” hyperglycemia and – more importantly – to erect these defenses inside the Stage 2-Stage 3 transition. Indeed, all the tDC embodiments studied and used to date to treat T1D, at least in the NOD/LtJ mouse model of the disease, have resulted in the complete prevention of hyperglycemia and varying success in “reversal” of hyperglycemia and maintenance of an insulin-free, stable, long-term, normoglycemic state (reviewed in (11, 12, 51–54) and listed in **Table 2**).

**Table 2:** Ex vivo approaches to generate tDC, phenotypes of the cells, and major effects.

GM-CSF?	IL-4?	IL-10?	TGFβ?	Other?	Ag provision?	DC phenotype	Effects	Reference(s)
YES	NO	YES	YES	NO	NO	↓Costimulatory molecules; ↓ IL-12, IL-23, IL-6; ↑ IL-10	Reduces insulitis. Prevents spontaneous diabetes in murine T1D models. Induces Tregs. Induces hyporesponsiveness of T-cells. Inhibits T-cell proliferation	(55, 56)
YES	YES	YES	YES	NO	NO	Intermediate expression of MHCII, CD40, CD80, CD86, CD83; ↓ IL- 12p70, IL-23, TNFα; ↑ IL-10, IL-6, PD-L1	Decreases T-cell peri-islet infiltration in rodents. Reduces T-cells Proliferation in rodents. Induces Tregs in rodents. Prolongs the survival of syngeneic Islet transplants in NOD mice	(57–59)
YES	YES	YES	YES	Insulin; GAD65	YES	↑ CD1a; ↓Costimulatory molecules (CD40, CD86); ↓ CD83; ↓ MHC-II, IL-12; ↓ IL-23; ↑ PGE	Suppresses effector/memory T-cells in rodent and human experiments. Induces T-cell anergy in rodent studies. Induces Tregs. Induces IL-10 production by T-cells in rodent and human cells. Suppresses T-cell proliferation. Induces hyporesponsiveness of rodent and human T-cells.	(60, 61)
YES	YES	NO	NO	Vitamin D+Dexamethasone+Pro-insulin	YES	↓ MHC-II, IFNγ, CD86; ↑ IL-10, PD-L1	Controls autoimmunity in rodent models. Induces Tregs. Inhibits effector T-cells. Eliminates CD8+ T-cells in rodent models.	(62, 63)
YES	YES	NO	NO	Vitamin D+Dexamethasone+GAD65	YES	↓Costimulatory molecules (CD40, CD86), CD83, MHC-II; ↑ CD14, TLR-2, PD-L1, IL-10; ↓ IL-6, TNFα, IL-23, IL-12p70	Decreases Th1/Th17 responses in rodent models. Suppresses antigen-specific T-cell activation and proliferation in rodent and human experiments. Prevents onset diabetes in NOD-SCID mice. Decreases IFNγ production by T-cells in rodent and human cultures.	(54, 64)
YES	YES	NO	NO	Rapamycin	NO	↓Costimulatory molecules (CD40, CD80), IL-6, IL-23; ↑ PD-L1	Induces Tregs. Inhibits T-cell proliferation in rodent experiments. Reduces Th17 cells in rodent experiments.	(57, 65)
YES	YES	NO	NO	Antisense DNA to primary transcripts of CD40, CD80, and CD86	NO	↓Costimulatory molecules (CD40, CD80, CD86), NO, TNFα, IL- 12p70	Prevents diabetes in NOD mice. Reduces insulitis. Promotes Tregs. Increases B-cells and Bregs in human and rodent cell cultures. Suppresses human and rodent T-cell proliferation:	(66–68)
YES	YES	NO	NO	Pro-insulin	YES		Delays or halts progressive destruction of β cell and loss function in mouse models.	(15)
YES	YES	NO	NO	Liposomes	YES	↓Costimulatory molecules (CD40, CD86); ↑ PDL1 expression, VEGF secretion	Arrests autoimmunity in rodent experimental diabetes	(69, 70)
YES	YES	NO	NO	TLR ligand	NO	↑ PD-L1, IRAK-M; Minimum increases of MHC-II, CD40, CD80, CD83, CD86	Suppresses T-cell activation and proliferation in rodent cell culture. Delays insulitis in NOD mice	(71)
YES	NO	NO	NO	Apoptotic bodies	NO	↓Costimulatory molecules (CD40, CD86), IL-6, TNFα	Reduces disease incidence in NOD mice. Reduces insulitis in NOD mice	(72)
YES	NO	NO	NO	Liposomes	YES	↑ TIM4, CD36; ↓ MHC-II, Costimulatory molecules (CD40, CD86); ↑ CCR7, CCR2, DC-SING; ↓ IL-6; ↑ Anti-inflammatory cytokines (IL-10, TGFβ1)	Decreases CD8+ T-cell proliferation in rodent cell model. Reduces disease incidence in NOD mice. Reduces insulitis in NOD mice.	(73)

## What are tolerogenic DC? the state of the current knowledge

DC exist as a spectrum of phenotypes and immune actions between pro-inflammatory and anti-inflammatory cells at the population and single cell level (reviewed in (11, 12) and **Table 3**). At one end of this spectrum, they are widely referred to as “mature” as a consequence of their response to entering inside, or being subjected to a building micro-environment of tissue “damage/danger” (74–82). Under such micro-environmental conditions, tissue-resident and/or migratory DC increase the expression of major histocompatibility complex (MHC) proteins loaded with peptides acquired from cells and proteins from their microenvironment, and upregulated the cell surface levels of co-stimulatory proteins like CD40, CD86, and a series of cell-cell adhesion molecules – some with signaling capacity into and inside from T-cells (83–86). DC undergoing maturation will begin migrating towards the lymphoid organs that drain the particular anatomic site carrying with them, and presenting acquired proteins via the MHC. As they mature, they produce a spectrum of pro-inflammatory immunokines which will be required to prime the T-cells whose T-cell receptor(s) “match” the peptide:MHC complex inside the lymphoid organ(s) (10, 87).

**Table 3:** Major phenotypes and actions of DC with inherent tolerogenic capacity.

DC population	Ag presentation ability	Co-stimulation capacity	Cytokine production	Inhibitory proteins	Anergy capacity	Clonal deletion of autoreactive T-cells	T-cell phenotypic skewing	B-cell skewing
Endogenous “immature”	Low Reduced class II MHC High DR/CLIP	Low to none (low CD40, CD40, CD86)	Low production of IL-12, IL-1β, TNFα, IL-6 (mouse and human)	Intermediate levels of FasL, PD-L1, ILT-3/4, CD39, CD73, IDO	YES	YES	TH2, Tr1, Foxp3 Tregs (mouse and human)	Unknown
Endogenous “semi-mature”	Reduced to intermediate levels of surface class II MHC (mouse and human)	Low to intermediate surface expression of CD40, CD80, CD86 (mouse and human)	Low production of IL-12, IL-1β, TNFα, IL-6 Intermediate production of IL-4, IL-10, TGFβ	Intermediate levels of FasL, PD-L1, ILT-3/4, IDO	YES	YES	TH2, Tr1, Foxp3 Tregs	Unknown
Ex vivo monocytic-derived tolerogenic	Reduced to intermediate levels of surface class II MHC (mouse and human)	Low to intermediate surface expression of CD40, CD80, CD86 (mouse and human)	Intermediate to high production of IL-4, IL-10, TGFβ (mouse and human)	Intermediate to high levels of FasL, PD-L1, IDO	YES	YES	TH2, Tr1, Foxp3 Tregs (mouse and human)	IL-10+ Bregs (mouse and human)

At the other end of the phenotypic spectrum are DC exhibiting immunosuppressive, tolerogenic ability *in vitro* and *in vivo* (54, 88–93). The shared characteristics of these tDC include a low capability to stimulate T cells and low to absent IL-12 production *in vitro*, expression and production of immunoregulatory molecules such as anti-inflammatory cytokines [IL-10 and transforming growth factor (TGF) β], indolamine 2,3- dioxygenase (IDO), and the expression of surface inhibitors like programmed death-ligand 1 (PD-L1) (89, 94). tDCs act at different levels [reviewed in (10–12, 54, 88, 90)]: a) they can directly delete autoreactive T-cells; b) they can purge autoreactive CD8+ T cells through a mechanism referred to as peripheral cross-tolerance; c) they can enforce clonal deletion and clonal anergy; and d) they can convert naïve, and in some instances effector, T-cells in the periphery into regulatory T cells (Tregs).

## Characteristics of tDC with tolerogenic potential

The ability of tDC to generate and to participate in establishing and maintaining tolerance inside the visceral and peripheral organs has been well-documented (38, 39, 43–49, 88, 89, 94). Migratory DC survey local and circulating antigens derived from the tissues into which they penetrate including the indigenous microbiota as well as foreign organisms such as viruses and bacteria (95–98). The antigens acquired from the cell constituents of these tissues as well as from the phagocytosis/endocytosis of the microbiota in the steady state are presented to T-cells inside the tissue-draining lymphoid organs under conditions where the presenting DC is in an immunologically “immature” or “semi-mature” state (92, 99–108) resulting in the induction and/or maintenance of T cell tolerance (10–12, 88, 89, 109, 110). Among the better understood mechanisms of how tDC achieve tissue- and organ-level immune tolerance to the tissue- and organ-restricted antigens is the induction and maintenance of Treg frequency and immunosuppressive stability. Peripheral tolerance as a function of tDC is partially-conditioned on the success of converting and maintaining T-cells into stable Tregs, many that express the Foxp3 transcription factor (54, 111–117). CCR7+ DC express a number of immunoregulatory molecules, including CD200, PD-L1 and PD-L2, facilitating tolerance *in vivo* (118, 119) even though it remains unclear if these DC facilitate the conversion of T-cells into Tregs or expand existing Tregs. In mice, migratory DC with tolerogenic potential carry acquired antigens from the parenchyma into the draining lymphoid organs, and in some cases even into the spleen (120, 121). While T-cells inside the lymphoid organs scan the arriving DC via TCR, another process is also active; where the antigens on the arriving DC are “cross-dressed” to tDC inside the lymphoid organs (10, 90, 122, 123). These antigens, irrespective of how they are delivered to the DC, can act as targets for T-cells that, in an environment of low pro-inflammatory signals, convert Tregs (90, 93, 124). This conversion is mainly driven by migratory and tissue-resident DC (that can migrate to the draining lymphoid organs) that have been positioned inside the family of Batf3+, XCR1+ type 1 subset (cDC1) (10, 90) as well as the more recently-described DC5 population which resemble plasmacytoid DC (125). Other distinguishing features of tDC include their expression of DEC-205, CLEC9A, BTLA (90, 124, 126–132). In addition to these characteristics, tDC induction of peripheral Tregs is dependent on their production of transforming growth factor β (TGFβ) which, alone or optimally together with DC-produced retinoic acid (RA) drives the expression of Foxp3 (127, 132). Others have reported that a subset of tDC expresses the enzyme indoleamine 2,3-dioxygenase (IDO), which prevents the expansion of effector T-cells by catabolism of tryptophan (133–143) and illustrated in **Figure 1**.

## How do tDC relevant in modifying T1D autoimmunity work?

There is no one specific mechanism of action through which tDC have demonstrated immune action and this is a significant advantage of tDC use for treatment of autoimmunity over single target, single mechanism approaches. **Figure 1** illustrates those that are better characterized. First, is the general characteristic of immature DC that exhibit low co-stimulation capacity (i.e. antigen:MHC presentation to T-cells in the absence of, or very low binding of T-cell CD28 with CD80/CD86 on DC). This results in the impaired ability of the responding T-cells to produce IL-2 and proliferate (144). Second, is the increased provision of inhibitory signals by the DC to the T-cells via programmed death-ligand 1 (PD-L1) (145–149), triggering the activation of SHP-1 and SHP-2 which intercede to induce clonal anergy and abrogated Treg differentiation (150, 151). Further, CTLA-4 expressed by activated T-cells and Tregs, can bind CD80 and CD86 on DC and facilitate their degradation (152). This additional mechanism of action by immature (and inherently tolerogenic DC) acts at another level to impair the priming of naïve T cells (153). Within the scope of action of CTLA-4, tDC can directly promote antigen-specific suppressive capacity inside CD4+ and CD8+ T cells with high CTLA-4 expression (154, 155). Finally, some populations of tDC can directly kill T-cells directly via Fas-FasL or TRAIL-mediated mechanisms (156, 157).

tDC express an array of immunoregulatory immunokines and metabolites that transform the local microenvironment into a stroma that facilitates conversion and/or stabilization of leukocytes, including T-cells and B-cells into immunosuppressive cells. Many tDC embodiments produce IL-10 (57, 158) and elicit anergy in effector and memory CD4+ T cells, at least *in vitro* in both mice and humans (58, 155). tDC also produce TGF-β and IL-27, with or without retinoic acid (RA) which convert T-cells into, or cause the proliferation of existing Tregs, into IL-10 producing regulatory T-cells that express Foxp3 or are characterized as an additional Tr1 population (159–165). It is important, at this time, to note that many of the study outcomes referred to inside this section have been obtained mostly in experiments where the tDC and other leukocyte populations, including antigen-specific T-cells and T-cells or B-cells that acquire a tolerogenic capacity, are brought together *in vitro*. Demonstration of physical interactions *in vivo* between tDC and T-cells and/or B-cells *in vivo*, inside affected tissues or the lymph nodes into which they drain to remains to be confirmed and is of high experimental priority.

## The tDC-B-regulatory cell system

In our phase I clinical trial to determine safety of tDC generated as monocyte progenitors in the presence of a mixture of antisense oligonucleotides targeting the primary transcripts of CD40, CD80, and CD86 (13), we discovered that these DC caused an increased frequency in circulating B-cells with characteristics shared with what were beginning to be referred to as “B-regulatory cells” (66, 67). This was the first in history report that tDC administration into humans could augment such cells and we further demonstrated that indeed B-cells are converted into Bregs and existing Bregs proliferate in the presence of tDC partly via retinoic acid (66). In a subsequent line of investigation, intravenous administration of vitamin D3-conditioned tDC reduced EAE in mice via Bregs (166).

Bregs were identified as a distinct population of immunosuppressive leukocytes in mice and humans and while they express IL-10, TGF-β, and IL-35 (167–182), these cytokines are not conditio sine qua non for their suppressive ability (183–186). In fact, IL-10-expressing B-cells can interact with DC in a contact-dependent manner in the NOD/LtJ mouse model of T1D conferring to them a regulatory capacity to suppress CD8+ diabetogenic T-cells (187). Bregs, remarkably, can inhibit the differentiation of T-cells into Th1, Th17, follicular helper T cells (Tfh), and the differentiation of B-cells into terminal B cells. They have been reported to also stimulate T cell anergy, expansion of Tregs, and to regulate the responses of invariant natural killer T-cells (iNKT) (181, 182, 188, 189). Vitamin D3-generated tDC have also demonstrated tolerogenic activity in the EAE mouse model of multiple sclerosis, where recipients exhibited clinical grade of the disease together with an increase in the frequency of Bregs (166).

These results reveal a second network of immunoregulatory leukocytes that can be upregulated and maintained by tDC to treat autoimmunity.

## Interweaving networks of tDC-orchestrated immune regulation

“Infectious tolerance” and “linked suppression” refer to processes where a signal that triggers localized immune hyporesponsiveness, once established, causes a local loop of immunoregulatory action and “programs” other local leukocytes toward a tolerogenic phenotype, promoting localized peripheral tolerance. While Tregs are key to this phenomenon (190), significant and substantial evidence strongly supports tDCs as the “programmers” of local imprinting of T-cells into Tregs and B-cells into Bregs (as well as stimulators of the proliferation of existing Tregs and Bregs), resulting in cell-mediated suppression of effector autoreactive T-cells, deletion of naïve autoantigen-specific T-cells and tDC imprinting of the environment into an immunosuppressive state via tDC production of IL-10, IL-35, and TGF-β. Indeed, tissue-resident DC as well as migratory DC can be imprinted away from a potential “maturation” and into a tolerogenic state inside such an environment, thus adding a third network of leukocytes under the control of tDC (191). For example, VitD3 tDC induce autoantigen-specific Tregs that can not only repress autoreactive T-cells via linked suppression, but can also program semi-mature DC towards a state of immunosuppression, at least *in vitro* (192). Retinoic acid induces tDC *in vitro*, which – on their own - produce retinoic acid promoting IL-10-producing Tregs, amplifying a local environment of immune hyporesponsiveness (193). Given the multi-directional interaction among tDC-Trtegs-Bregs-and programming of other DC towards a state of tolerance induction potential, the process of “infectious tolerance” and “linked suppression” is established inside the lymphoid organs that drain tissues that are targets of autoimmunity and/or inside the autoimmunity target tissues themselves. This might explain why systemic administration of immunosuppressive cytokines, like IL-10, have not always resulted in efficacious treatment of autoimmunity; local IL-10 increase would maintain survival and stability of a network of tDC : Treg:Breg:imprinted DC. This would constitute a “tolerogenic feedback loop” which initiates and maintains a steady state of localized immune tolerance (194). Re-establishment of such a network is one of the bases of tDC therapy that we, and others, aim to achieve and stabilize in T1D.

## Current methods to generate tDC relevant to modify T1D autoimmunity

By convention, DC characterized as “immature” or “semi-mature” cause immune hyporesponsiveness including antigen-specific immune hyporesponsiveness *in vitro* and *in vivo* (11, 12). Oftentimes, this translates into a tolerogenic capacity at the functional level. A specific population of DC with tolerogenic properties has been identified in humans (158) and these DC promote immune hyporesponsiveness in allogeneic CD4+ T cells, produce IL-10, and express the surface markers CD163, CD141, CD16, and CD14 (158). While their ontogeny is unknown, it is very possible that many more DC populations with tolerogenic phenotypes may exist and that this – as well as others waiting to be discovered - could in fact represent transient states captured as a consequence of time at sampling. Additionally, and more excitedly, these may be bona fide steady state tDC whose isolation and expansion would be a tremendous leap in tDC treatment of autoimmunity and transplantation tolerance induction.

While purity markers remain to be discovered for such DC populations, and given the phenotypic instability of “immature/semimature DC” *in vivo* (195), a number of methods have been developed to generate tDC *in vitro* and *ex vivo*, mostly based on monocytic progenitors (196). A variety of cell culture methods, shown in **Table 2** and illustrated in **Figure 1** have achieved cells with tolerogenic actions, underlied by mechanisms that involve Treg induction/preservation (196, 197).

The basis of generating tDC *in vitro* and/or ex vivo from monocytic progenitors rests on the discovery, more than 25 years ago, that GM-CSF and IL-4 caused the differentiation of monocytes into “immature” DC. These cells are inherently tolerogenic, eliciting T-cell immune hyporesponsiveness (92). These DC express low surface level class II MHC, CD80, and CD86 and by their low co-stimulation ability, they were shown to protect NOD/LtJ mice from T1D as they expressed IL-10 and dampened CD8+ effector function (116, 198–200). tDC were also generated from monocytic progenitors in the presence of IL-10 and TGFβ and these DC elicited antigen-specific immune hyporesponsiveness as well as efficient induction of anergy and generation of Tregs (58). tDC have been generated from monocytes only in the presence of IL-10 (57, 58) or TGFβ (59). These DC exhibit increased production of IL-10 and IL-6, reduced IL-12p70 production, and a “semi-mature” phenotype (expression of class II MHC, CD40, CD80, and CD86, at levels between “immature” and “mature” DC). Another GM-CSF-based method to generate tDC from monocytic progenitors involves the addition of IL-10 with the result that these DC can attenuate insulitis and spontaneous diabetes development in NOD/LtJ mice. These DC have also demonstrated efficacy in long-term survival of allogeneic islets of Langerhans transplants, but act in an antigen non-specific mechanism of action (55). In a similar manner, TGFβ-generated DC from monocytic progenitors also enhanced the survival of allogeneic islet transplants into autoimmune recipient diabetic mice (59).

As concerns the use of tDC to treat T1D, two DC embodiments have passed initial safety outcome measures and on the threshold for phase II trials; those generated with GM-CSF/IL-4 and a mixture of antisense oligonucleotides targeting the primary transcripts of CD40, CD80, and CD86, and those DC generated in the presence of 1,25-dihydroxyvitamin D3 and dexamethasone. While the former tDC are locked into a state of low/absent co-stimulation *in vivo* in a stable and long-term manner due to modification of transcription/translation (13, 52), the latter also exhibit low co-stimulation capacity (53). These cells exhibit high CD11c, DC-SIGN, PD-L1, and low class II MHC expression, supporting a state of “semi-mature” phenotype. Additionally, they also produce IL-10 (65, 201–203). A comparison of tDC generated with vitamin D3, IL-10, dexamethasone, TGF-β, or rapamycin demonstrated common features: low co-stimulation potential, and production of IL-10, conditions optimal for the induction of peripheral Tregs (57). **Table 2** lists other approaches that resulted in shared phenotypes along the line of low co-stimulation potential (58, 196). Even though the maturation state of the tDC may not always distinguish functionally-tolerogenic DC (204), one additional feature that should be considered important towards tolerance capacity is the DC ability to migrate towards the lymphoid organs draining a tissue/organ targeted in an autoimmunity. This can explain the instances where therapeutic benefit was achieved in the absence of provision of disease-specific antigen(s) to the DC *in vitro* or *ex vivo* (57, 205).

## Harmonizing tDC characteristics relevant for modifying T1D autoimmunity

A few years ago, an important publication offered some recommendations on what to consider as pre- and co-clinical measurements of tDC stability, potency, and stability (110). For example, the increased frequency of highly-suppressive Tregs generated in co-culture with IL-10-conditioned tDC (57, 205), restrained proliferation and activation of autoreactive CD4+ T-cells in the presence of 1,25-dihydroxyvitamin D3-conditioned tDC *in vitro*, and IL-10 expression in T cells are all hallmark capacities of tDC (206). Additional “readouts” of tDC can be Breg expansion (either expressing IL-10 or not) as well as their differentiation inside B-cell culture (67). Breg expansion/differentiation by tDCs is partly mediated by their production of retinoic acid, which incidentally, on its own, also causes the differentiation of Foxp3+ Tregs (66). *Ex vivo* generated tDCs with impaired costimulatory capability, delay and “reverse” new-onset hyperglycemia in association with increased frequency of Tregs in diabetes-free recipients (67, 68).

tDC with low co-stimulation potential have been mobilized effectively whether they are further modified by loading of specific Ag or not. Proinsulin, insulin, and GAD65 have been used alone or in combination as Ag added to tDC prior to cell administration *in vivo*. Vitamin D3/dexamethasone-generated and proinsulin-loaded tDC induce antigen-specific Tregs with various phenotypes *in vitro* (e.g. cell surface Lag-3, CD161, and inducible co-stimulator). These Ag-loaded tDC can suppress effector CD8+ and CD4+ T cells (62). In a humanized mouse model of T1D, the administration of tDC loaded with proinsulin controlled the progression of T1D, possibly via IL-10 production (63). In another study, vitamin D2/dexamethasone-generated GAD65-loaded tDCs were shown to prevent the adoptive transfer of diabetes by diabetogenic splenocytes to NOD-SCID recipients. However, in this study the Ag-loaded tDCs were not as effective as the parental non-Ag DC in protecting from T1D (64) supporting the model where Ag is acquired endogenously inside the lymph nodes draining the inflamed pancreas and where no *ex vivo* Ag loading is necessary.

While Ag loading of DC may or may not enhance the tolerogenic potential, treatment *ex vivo* with apoptotic target tissue may be an alternative method to provide a wide array of Ag to the tDC. In T1D, the increase in apoptotic pancreatic β-cells or defects in the process of capture and phagocytosis of apoptotic bodies (referred to as efferocytosis) contributes to the loss of tolerance (207). DC acquire a tolerogenic phenotype and functionality after ingestion of apoptotic β-cells and prevent T1D when transferred to NOD mice (72). The limitation of availability of freshly-collected human pancreas apoptotic bodies, however, necessitates different vehicles to promote DC efferocytosis. One such alternative are phosphatidyl-serine liposomes containing β-cell autoantigens and these were able to arrest autoimmunity and prevent T1D through the generation of tDCs. Liposome-exposed DC slowed the proliferation of autologous T cells, interfered with antigen presentation, and increased the expression of genes associated with a phenotype of tolerogenic state as well as anti-inflammatory pathways (69). Additionally, insulin-loaded liposome administration into NOD/LtJ mice reduced the severity of insulitis and expanded antigen-specific CD4+ T cells (73). Modulation of pattern recognition receptor signaling has been shown to be an innovative method to generate tDC [65]. DC exposure to a polyethylene glycol-conjugated TLR-7 ligand followed by the administration of these DC into NOD mice delayed the onset of T1D and insulitis (208). TLR-2 activation with its agonist, Pam3CSK4), resulted in DC-dependent suppression of T-cell activation (209) and combining this with a co-treatment with a dipeptidyl peptidase 4 inhibitor, which increases the mass of β-cells, “reversed” hyperglycemia in newly-diabetic NOD/LtJ mice (71).

## Autoantigen- or auto-antigen-derived peptide-pulsing: is it required for Ag-specific tDC?

The underlying cause of tissue- and organ-specific autoimmunity is the failure of central tolerance, where AIRE+ medullary thymic epithelial cells together with medullary dendritic cells do not express autoantigens at levels adequate to present to thymocytes. As such, every tissue- and organ-specific autoimmunity has its specific spectrum of autoantigens together with a spectrum of thymocytes that react to those autoantigens – and that escaped the thymus. Some studies have shown that pulsing tDC with these autoantigens confer antigen specificity to immunosuppression by permitting those DC to be scanned by T-cells whose TCR are specific for that autoantigen:MHC complex (64, 105, 200). It stands to reason that this approach would be more effective in silencing autoreactive T-cells, minimizing the potential of restraining/anergizing T-cells that are not autoreactive. Where the autoantigens are well-characterized, they can be used in combinations to pulse tDC during or following *ex vivo* DC generation. GM-CSF+IL-4, dexamethasone (or Vitamin D3) tDC pulsed with myelin autoantigens have shown efficacy in treating multiple sclerosis in mouse models of disease (54). For rheumatoid arthritis, DC generated in the presence of GM-CSF+IL-4, and then supplemented with the NF-κB inhibitor Bay 11-7082, subsequently pulsed with citrullinated peptides of aggrecan, vimentin, collagen type II, and Aα and Bβ fibrinogen (putative RA autoantigens) conferred superior efficacy *in vivo* (210, 211). Given the heterogeneity of the autoimmunity progression, self-antigens are not necessarily universal. Different patients can exhibit T-cells reactive to different autoantigens. In T1D, different patients exhibit auto-antibodies different in antigen specificity and this is also seen in T-cell reactivty as well. The spectrum of autoantibodies and autoreactive T-cells also changes in the progression of the disease and different patients exhibit different changes in this progression (212–215). While insulin and GAD65 are the major T1D auto-antigens, rationally positioning them as the two key autoantigens to pulse tDC with in order to confer antigen-specific tolerance, as the autoimmunity progresses, T-cells reactive to other β cell proteins appear in the circulation, thus reflecting the process of antigen spreading. With disease progression, the autoimmunity would now be driven by the expanded set of autoantigens. Therefore, restricting the choice of autoantigens to reflect the common two (insulin and/or GAD65) could reduce the effectiveness of tDC treatment. This has already been demonstrated in T1D (200). A pre-emptive approach could be to serially-ascertain autoantigen profiles (e.g. serial assessment of T-cell reactivity to a panel of known autoantigens) in a patient-specific manner. This, although expensive, could improve antigen specificity in tDC-based tolerance, but would still not guarantee a complete coverage of all possible autoantigens (the list of all possible diabetes antigens remains under investigation as a growing number are post-translational modifications of previously-unexpected proteins (216–221). An alternative, autoantigen-agnostic, patient-specific approach could be informed by pivotal studies in the use of tDC for rheumatoid arthritis. Here, synovial fluid from inflamed joints of each patient was added to the cultured tDC prior to administration. The supposition was that all, possible autoantigens would be present inside that fluid (222, 223). Adaptation of this approach for T1D however is not feasible, as a patient-specific tailoring of tDC would require islet-containing pancreatic tissue resection from the patient. Another important consideration is based on the choice of the leading phase I clinical studies using tDC for T1D to administer the cells into an abdominal region that drains, partly, into the pancreatic lymph nodes (13, 14). An Ag-agnostic approach, i.e. to allow the Ag draining to those same lymph nodes from the pancreas, is a more natural process to allow the exogenously-supplied tDC to acquire those Ag. Recent studies have demonstrated that, even during the steady state, extracellular vesicles containing β cell Ag drain into the pancreatic lymph nodes (224). Under inflammatory conditions, such as autoimmunity, the rate of drainage is expected to increase resulting in an increase of uptake of these Ag by exogenously-supplied tDC which also drain inside these organs. As part of the process, these vesicles can also be aquired by lymph node-resident DC as well as by migratory DC that accumulate inside the pancreatic lymph nodes. By cross-presentation and/or cross-dressing, these Ag will be acquired by the tDC. This, we believe, is the basis for why our approach as well as those of others who have not “pulsed” tDC with specific Ag, have demonstrated outcomes supportive of a therapeutic benefit (13, 52, 198).

## Human clinical trials: where are we now?

Our team in Pittsburgh was the first to historically demonstrate that tDC were safe and well-tolerated in adult insulin-requiring adult T1D (13). Remarkably, we detected C-peptide in 4/7 recipients of the tDC product and in one of the recipients of the parental GM-CSF/IL-4 DC. The observation of an increased frequency of B-cells inside which were elevated numbers of Bregs peri-treatment led us to demonstrate, in the NOD/LtJ mouse strain that DC directly promoted B-cell differentiation towards Bregs and existing Breg proliferation, partly *via* a retinoic acid-based mechanism (66, 67). In 2018, the NIDDK TrialNet Consortium approved a phase II study with these tDC in newly-diagnosed T1D participants, however at that time, NIDDK stopped supporting the production of study agents used in TrialNet studies. Thus, this phase II study, referred to as “TN-24”, even though it remains, will require some other support to generate the cell products. Work is underway to realize this over the next 12 months (Clinicaltrials.gov identifier: NCT02354911).

Where several pre-clinical studies have demonstrated T1D Ag-loaded tDC to induce Ag-specific tolerance, administration of pro-insulin peptide loaded tDC, while safe and well-tolerated, did not achieve any remarkable changes in C-peptide post-baseline (15). While this may appear disappointing, it is important to note that the trial was conducted in long-standing T1D patients, while Ag-loaded tDC are more effective in newly-diagnosed situations. Also, and of importance to all immunomodulation approaches in T1D, including the recently-approved Teplizumab, the extent of the progression of the inflammation as well as the impact of uncontrolled glycemia, uncompensated state and activation levels/numbers of β cell-targeting lymphocytes at the time of treatment, could be important determinants of effectiveness and efficacy (54, 60, 67). Additional points of consideration in tDC therapy include: a) Stability of the tDC once administered *in vivo*; b) progression of the disease state at the time of tDC administration; c) effect of T1D *endotypes* (225) on disease progression and response to tDC – this informing that tDC may be effective only in individuals exhibiting certain endotypes and at certain points in disease progression. In line with these determinant, a phase I study has been proposed to test pro-insulin peptide-loaded tDC in T1D participants who use insulin and do not exhibit any co-morbidity or other health condition (clinicaltrials. gov identifier: NCT04590872).

## A side note: serial co- or alternating administration of autologous tDC and Tregs; lessons from the field of tumor immunotherapy

A number of tumor immunotherapy approaches involve ex vivo generation of tumor-specific T-cells generated in the presence of tumor antigen-pulsed DC ex vivo and administration of the expanded T-cells (226–229). This approach can be adapted for T1D leukocyte co-therapy as we describe herein. Ex vivo generation of Tregs is a technical reality and administration of autologous Tregs for T1D has shown some degree of efficacy (230–233). Emerging evidence indicates that these Tregs do not necessarily home to the pancreas and that their half life is not particularly well-characterized, especially inside the pancreas (230, 234). Another point of remaining interest is the capacity of these Tregs to directly suppress autoreactive T-effectors, or their participation in networks where other cells are indispensable to carry out the final acto of suppressing autoreactive lymphocytes. IL-2 supplementation *in vivo* may provide some degree of stability for the Treg half life (NCT02772679), however CD25 surface levels may limit the effect of the cytokine (235). In spite of these known or proposed limitations, Treg therapy remains under clinical investigation in T1D (CLBS03; NCT02691247) as well as in lupus (NCT02428309) and autoimmune hepatitis (NCT02704338)

Co-administration or serial/alternating administration of tDC with Tregs therefore becomes a question of clinical interest, especially at the time of new-onset T1D. The inter-relationship of tDC and Tregs facilitates inter-dependent stability of what we propose will be a very stable and robust network of peripheral tolerance. Serial/alternating administration, or co-administration of tDC and Tregs would stabilize Foxp3 expression and the stabilized Tregs would in turn reinforce the tDC tolerogenic state via cell-cell interactions and immunoregulatory cytokines acting in a paracrine manner. We envisage and propose an initial co-administration of tDC and Tregs followed by periodic “boosters” of serially-alternating tDC and Tregs. Or even serially co-administered cells. Logistically, we do not anticipate generation of tDC and Tregs in parallel from the same leukapheresis product technically challenging. From the common leukapheresis product, the same cell generation GMP facility could, in parallel, generate the tDC from the monocytic precursors as well as the Tregs from the PBMC population. At least pre-clinically, we believe the time is now to test this approach, certainly for T1D.

## Clinical protocol design considerations in detecting efficacy in phase II studies

While the fundamental CMC among the currently clinically-considered tDC rely on GM-CSF and IL-4, there is a divergence in the remaining aspects of tDC generation and administration int humans. These differences could be the basis of efficacy/failure, different levels of efficacy – outcomes that are dependent on different mechanisms of action once the cells are administered. One important variable that might affect efficacy is cell dose (least number of cells and frequency of administration to achieve significant efficacy). Another variable is the administration site itself. We believe that this second variable is particularly important as the target site (inflamed anatomic region) of different autoimmunities is subserved by different anatomic points of entry for exogenously-introduced cells. There is a growing consensus that the ideal point of entry of exogenously-administered tDC is an anatomic site draining to lymphatics that also drain the autoimmunity target organ/tissue. Lymph nodes that drain the target tissue of autoimmunity often exhibit high concentrations of activated autoreactive T-cells (236). Examples include the cervical lymph nodes, targeted for tDC-based treatment of multiple sclerosis (clinicaltrials.gov identifier: NCT02618902) and the pancreatic lymph nodes for T1D, as we have demonstrated (13). Alternatively, if a target site of autoimmunity is well defined, a direct access to administer the tDC can be feasible as shown for Crohn’s disease (237).

As tDC are more widely-accepted for treating autoimmune diseases, some other variables for CMC harmonization should also be considered. For example, most, of not all the tDC preparations that are administered are not completely homogeneous; there are variable (albeit low) concentrations of “contaminating” undifferentiated or uncharacterized monocytic and granulocytic cells, carried over from the elutriation process. These carried-over cells could modify the tolerogenic capacity of the differentiated tDC once administered *in vivo*. Methods for further enrichment, removal of these carried-over cells should be pursued. A series of surface markers that identify only tolerogenic cells remains to be defined. It is also all but certain that a single dose of tDC will not result in stable remission of disease but a successful outcome will be a function of multiple administrations over time, at least in most individuals. Methods to expand an initial large batch of tDC for aliquoting into individual doses, stable to cryopreservation, without altering the phenotype and tolerogenic capacity of the individual aliquots, once thawed from cryopreservation, is an immediate CMC need. It remains unknown if thawed cryopreserved tDC are functionally-identical *in vivo* to the freshly-obtained cells. In this regard, international collaborations such as those that resulted in the first set of proposals for harmonization of tDC and Treg (Minimum Information about Tolerogenic Antigen-Presenting cells; MITAP) should be a priority and sharing of tDC generation protocols to verify and validate outcomes is critical (110).

## Disease stage and patient-specific modifiers of tDC efficacy

Some of the mapped T1D-associated genetic polymorphisms have been shown to affect DC function (238). The state of activation of effector T-cells together with the stability of the network of tolerogenic leukocytes at the time of tDC administration could conceivably affect the efficacy outcomes, either in delaying the progression across Stage 3 and/or “reversal” of newly-diagnosed hyperglycemia. This information is currently not well-developed and can affect the *in vivo* stability of tDC, as well as their ability to strengthen weakened regulatory networks and/or delete/attenuate effector lymphocytes once administered into study subjects. For example, even as IL-10/TGFβ-generated tDC effectively induces insulin-specific tolerance in autologous effector/memory CD4+ T cells derived from T1D individuals, the degree of tolerance induction was dependent on the initial T-cell activation state of each study participant (60). These results were congruent with those of another study with IL-10/TGF-β-generated, insulin, or GAD65-loaded tDC from T1D patients (61). Furthermore, the variation of the differences in the evolving dysglycemic state among Stage 3 individuals as well as the newly-developed hyperglycemic state among T1D individuals, could affect the tolerogenic abilities of DC generated from their monocytic progenitors (239). For example, vitamin D2/dexamethasone-generated DC loaded with GAD65 from T1D patients exhibiting good or suboptimal glucose control exhibited significant variation in their ability to regulate TH1 and TH17 responses together with the suppression of antigen-specific T-cell activation, *in vitro*. When transferred into NOD-SCID mice, the tDC from these individuals, also exhibited a variability in diabetes delay dependent on the state of glucose control of the subject at the time of blood procurement (to generate the DC) (54). An understanding of genetic and metabolism-dependent differences in tDC activity and stability as well as the state of immune activation of the recipient at the time of tDC administration is therefore of some importance to dissect in the near future.

Irrespective of which tDC embodiment will be the finalist to demonstrate the most effective and resilient prevention of T1D and/or “reversal” of hyperglycemia, one critical point that must be taken into consideration is the resiliency and stability of the tolerogenic state of the DC once administered *in vivo*. The tolerogenic state and functions may not be guaranteed, especially should potently-inflammatory event arise at the site of tDC accumulation (e.g. viral infection). That is why, in our opinion, it is critical to ensure that any approach to generate tDC *ex vivo* considers ensuring a long-term stability of the tolerogenic state once the DC are administered. It is with this thought in mind that we began our investigations to generate tDC, targeting co-stimulation, more than 20 years ago (68).

## Conclusion

With the announcement of the approval of Teplizumab as a treatment to delay the progression of Stage 3 dysglycemia to overt, insulin-requiring diabetes, the media is promoting “the end of type 1 diabetes”. A simple examination of the data using Teplizumab over two decades, indicates otherwise. Furthermore, all the other alternative immunomodulation approaches to delay the disease process and/or “reverse” hyperglycemia have either failed, or exhibit minimal efficacy – in specific subpopulations of patients, and/or are associated with significant toxicity limiting their use and broad consideration (240–246). tDC are different and substantially promising for the following reasons: a) they act to generate Tregs and Bregs concurrently; b) they can induce other DC inside the environment they accumulate into to acquire tolerogenic capacity; c) They imprint a tolerogenic environment inside which they accumulate; and d) they are safe and well-tolerated in many human phase I studies in the space of autoimmunity [reviewed in (12)] We believe that either alone or potentially in concert with Tregs, tDC will surmount what is more of a conceptual rather than a practical barrier that currently has stalled their consideration in phase II studies. While phase II studies in rheumatoid arthritis are closer to realization than in T1D, we are very confident that the time is nearing where the first phase II study in T1D will be announced.

## Author contributions

NG wrote the original draft of the manuscript, edited all versions, and assumes responsibility of the final submitted draft.
